# The sequence and *de novo* assembly of the wild yak genome

**DOI:** 10.1038/s41597-020-0400-3

**Published:** 2020-02-24

**Authors:** Yanbin Liu, Jiayu Luo, Jiajia Dou, Biyao Yan, Qingmiao Ren, Bolin Tang, Kun Wang, Qiang Qiu

**Affiliations:** 1 State Key Laboratory of Grassland Agro-Ecosystems, School of Life Sciences, Lanzhou University, Lanzhou, China; 2 Research Center for Ecology and Environmental Sciences, Northwestern Polytechnical University, Xi’an, China

**Keywords:** DNA sequencing, Genome, Zoology

## Abstract

Vulnerable populations of wild yak (*Bos mutus*), the wild ancestral species of domestic yak, survive in extremely cold, harsh and oxygen-poor regions of the Qinghai-Tibetan Plateau (QTP) and adjacent high-altitude regions. In this study, we sequenced and assembled its genome *de novo*. In total, six different insert-size libraries were sequenced, and 662 Gb of clean data were generated. The assembled wild yak genome is 2.83 Gb in length, with an N50 contig size of 63.2 kb and a scaffold size of 16.3 Mb. BUSCO assessment indicated that 93.8% of the highly conserved mammal genes were completely present in the genome assembly. Annotation of the wild yak genome assembly identified 1.41 Gb (49.65%) of repetitive sequences and a total of 22,910 protein-coding genes, including 20,660 (90.18%) annotated with functional terms. This first construction of the wild yak genome provides a variable genetic resource that will facilitate further study of the genetic diversity of bovine species and accelerate yak breeding efforts.

## Abstract (summary)

**Table:** 

Measurement(s)	DNA • genome • sequence_assembly • sequence feature annotation
Technology Type(s)	DNA sequencing assay • sequence assembly process • sequence annotation
Sample Characteristic - Organism	Bos mutus
Sample Characteristic - Environment	Qinghai-Tibetan Plateau

Machine-accessible metadata file describing the reported data: 10.6084/m9.figshare.11778669

## Background & Summary

The yak, which can survive extremely cold, harsh and oxygen-poor conditions, is endemic to the Qinghai-Tibetan Plateau (QTP) and adjacent high-altitude regions^1^. Yak were domesticated by nomadic people from wild yak at least 7300 years ago^2^. Nowadays, more than 22 million domestic yak (*Bos grunniens*) provide necessities, such as food, transport, shelter and fuel, for Tibetans and other humans in high-altitude areas^1^. In addition, there are still 15–20 thousand wild yak (*Bos mutus*) surviving in northwestern parts of the QTP^3,4^. Due to long-term over-breeding and inbreeding caused by traditional yak breeding practices, the reproductive capacity, growth rate, adult size and milk production of domestic yak have declined and mortality has increased, especially among the newborn and young^1^. However, Datong yak, the only artificially cultivated yak breed that is a cross between wild yak and domestic yak, shows excellent growth characteristics and production performance. Datong yak are generally 30 and 50% heavier than domestic yak at birth and six months of age, respectively, produce more than 15% milk, and have 25 and 31% higher carcass weights at ages of 6 and 18 months, respectively^5,6^. The high growth, development and production rates of the Datong yak show the feasibility of improving traits of domestic yak with wild yak resources, and potential importance of exploiting wild yak genetic resources for yak breeding in the future. However, the wild yak genome has not been previously sequenced, which has impeded both research and breeding efforts.

Thus, to elucidate genomic features of this vulnerable species, we have constructed a draft genome for wild yak. We extracted genomic DNA from blood tissues, constructed 3 Paired-End (PE) and 3 Mate-Pair (MP) libraries, which were sequenced using the Illumina HiSeq. 2000 platform. After quality filtering and trimming of raw data, Genome Characteristics Estimation (GCE, v1.0.0)^7^ software was employed to evaluate the genome size using PE reads, and Platanus v1.2.4^8^ to assemble the genome using all clean data. In addition, GapCloser v1.12^9^ was used to perform another round of gap closure based on the assembly results. The final genome assembly size was 2.83 Gb, containing 808,541 contigs (N50 = 63.2 kb) and 734,073 scaffolds (N50 = 16.3 Mb), representing 91.5% of the estimated genome. Structural annotation of the genome yielded 22,910 genes, 90.18% of which could be functionally annotated with at least one of the five protein databases (TrEMBL, SwissProt, InterPro, GO and KEGG). The wild yak genome assembled in this study provides a valuable genetic resource for future efforts to protect the vulnerable wild yak and further comparative analysis of genome biology among bovine species to promote breeding research.

## Methods

### Sample collection, library construction and sequencing

Genomic DNA was extracted from a blood sample collected from a female wild yak originally captured from the wild and reared at the Datong Yak Farm of Qinghai Province (37°15′35.6″N, 101°22′24.0″E, altitude around 3200 m) using a standard phenol/chloroform method. The quality and integrity of the extracted DNA were checked by measuring its A260/A280 ratio and agarose gel electrophoresis. For paired-end libraries with insert sizes of 280, 500 and 800 bp, 6 μg portions of genomic DNA were used to generate the corresponding libraries using Illumina TruSeq DNA Nano Preparation Kit (Illumina, San Diego, CA, USA). For mate-pair libraries with insert sizes of 2, 5 and 10 kb, 60 μg portions of DNA were used for circularization and further library construction using Nextera Mate Pair Library Preparation Kit (Illumina, San Diego, CA, USA). Both the sample collection and experimental library construction protocol were approved by the Ethical Committees of Lanzhou University. All libraries were sequenced on an Illumina HiSeq. 2000 platform with 150 bp read length, following the manufacturer’s instructions. Finally, 760.85 Gb of raw data were generated in total (Table 1).

**Table 1:** Summary statistics of wild yak sequenced reads.

Library Insert Size (bp)	Raw reads			Qualified reads		
	Total Data (Gbp)	Reads Length (bp)	Sequence coverage (×)	Total Data (Gbp)	Reads Length (bp)	Sequence coverage (×)
280	106.81	150.00	35.60	103.96	145.26	34.65
500	89.10	150.00	29.70	86.03	144.27	28.68
800	109.24	150.00	36.41	102.80	140.32	34.27
2,000	117.87	150.00	39.29	108.44	150.00	36.15
5,000	166.14	150.00	55.38	147.18	150.00	49.05
10,000	171.69	150.00	57.23	113.89	150.00	37.96
Total	760.85	—	253.62	662.30	—	220.77

### Preprocessing and genome size estimation

All the sequencing reads were preprocessed for quality control and filtered with stringent criteria using Lighter v1.1.1^10^ software. Firstly, raw data were filtered by removing reads with >10% unknown bases. Then, paired reads with low-quality bases (quality scores ≤7) covering more than 65% of the read length were filtered out. Reads with PCR duplicates or adapter contamination were also removed. Finally, both read 1 and read 2 files were filtered out if they had >10 bp overlap, allowing 10% mismatch. In total, 662.3 Gb of clean reads were obtained after filtering (Table 1).

**Fig. 1:**
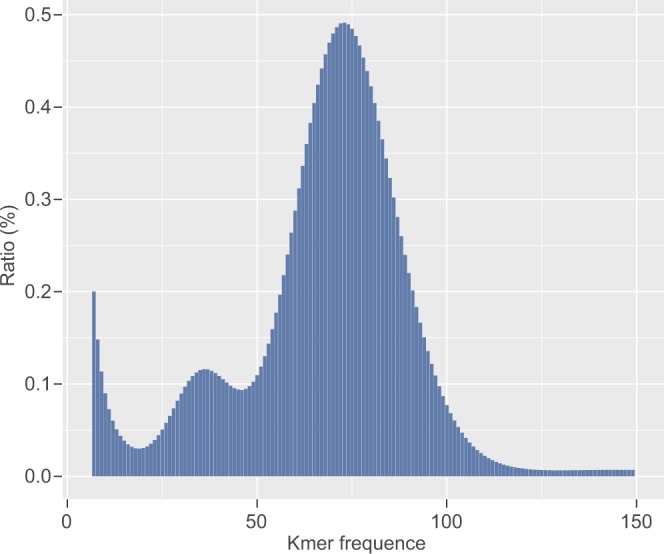
21-mer distribution in the wild yak genome.

Prior to genome assembly, all the preprocessed sequences from the short-insert library were subjected to genome size estimation using Genome Characteristics Estimation (GCE) with a *k* value of 21. The genome size of wild yak was estimated to be around 3.09 Gb, using the following formula: genome size = *k*-mer number/*k*-mer depth, where the *k*-mer number refers to the total number of *k*-mers, and *k*-mer depth is the depth of the main peak in the *k*-mer frequency distribution (Fig. 1).

### Genome assembly

For *de novo* genome assembly, Platanus software was used for constructing contigs and scaffolds with default parameters, and GapCloser was employed to fill the remaining gaps in the scaffolds with all sequencing reads. These steps finally yielded a wild yak draft genome with a total length of 2.83 Gb, accounting for 91.5% of the estimated genome size (contig and scaffold N50 sizes: 63.2 kb and 16.3 Mb, respectively) (Table 2).

**Table 2:** Summary statistics of the wild yak genome assembly.

	Contig		Scaffold	
	Size (bp)	Number	Size (bp)	Number
N90	8,406	52,665	418,892	355
N80	22,366	33,612	3,980,782	155
N70	35,157	23,897	6,690,007	102
N60	48,527	17,241	12,581,129	71
N50	63,194	12,274	16,301,239	50
Longest	811,040		75,900,441	
Total Size	2,751,522,574		2,831,279,091	
Total Number (>100 bp)	808,541		734,073	
Total Number (>2 kb)	83,689		19,451	
GC content			1,154,582,425 (41.96%)	

To evaluate the completeness of our assembly, we carried out BUSCO^11^ analyses and the results indicated that 3,974 of the 4,104 conserved single-copy genes in mammals were present in our assembly, of which 3,799 were single, 55 were duplicated, and 120 fragmented matches (Table 3). To validate the single-base accuracy of the genome assembly, we aligned the high-quality reads of short-insert libraries to the assembly using Burrows-Wheeler Aligner (BWA, v0.7.15-r1140)^12^ software, and the alignment outputs were converted to Binary Alignment Map (BAM) format via SAMtools v1.3^13^. The genome coverage was then calculated by a custom Perl script, which indicated that more than 93.9% of the assembly had >20-fold coverage.

**Table 3:** Summary of BUSCO analysis results: matches to 4104 single-copy orthologs in mammalia_obd9.

BUSCO mode	Species	Complete one-to-one match to ortholog	Complete match of multi gene copies to ortholog	Fragmented match to ortholog	Total number of matches to ortholog	No match to ortholog
Genome	Wild yak	3792 (92.4%)	59	123	3973 (96.8%)	130
	Domestic yak (version 1.1)	3809 (92.8%)	32	138	3979 (97.0%)	125
	*Bos taurus* (UMD3.1)	3794 (92.4%)	53	124	3971 (96.8%)	133
	Wisent (version 1.0)	3763 (91.7%)	31	180	3979 (96.8%)	130
OGS	Wild yak	3821 (93.1%)	70	119	4010 (97.7%)	94
	Domestic yak (version 1.1)	3987 (97.2%)	27	59	4073 (99.2%)	31
	*Bos taurus* (UMD3.1)	4009 (97.7%)	24	50	4083 (99.5%)	21
	Wisent (version 1.0)	3840 (93.6%)	63	165	4068 (99.1%)	36

### Repeat annotation

**Fig. 2:**
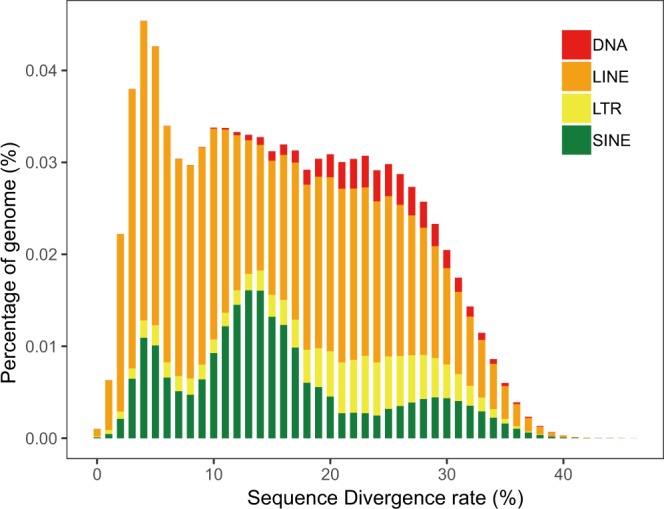
Sequences divergence rate of repeats annotated by RepeatMasker in wild yak. The x-axis represents the sequence divergence rate of repeats. The y-axis represents the percentage of repeat sequences in the genome.

Repetitive regions of the wild yak genome were identified using a combination of *de novo* and homology-based approaches, as applied in a previous analysis of the *Ovis ammon polii* genome^14^. For the *de novo* prediction, RepeatModeler v1.0.11 was employed first to construct a *de novo* repeat library, then RepeatMasker v4.0.7^15^ was used to identify repeats using both the RepBase^16^ library of known transposable elements (TEs) and a self-trained repeat database. Next, we applied RepeatProteinMask (a package in RepeatMasker) to identify repeats at the protein level using the TE protein database. In addition, tandem repeats were further annotated using Tandem Repeat Finder (TRF, v4.0.9)^17^. Finally, the non-redundant repeats were checked according to their coordinates in the genome. Overall, we identified 1.41 Gbp of non-redundant repetitive sequences, representing 49.65% of the wild yak genome assembly; of which long interspersed elements (LINE) were the most abundant, accounting for 35.98% of the whole genome (Fig. 2; Table 4).

**Table 4:** Summary statistics of interspersed repeats in the assembled wild yak genome.

Type	Repbase TEs		TE proteins		*De novo*		Combined TEs	
	Length (bp)	% in genome	Length (bp)	% in genome	Length (bp)	% in genome	Length (bp)	% in genome
DNA	58,420,396	2.06	2,646,996	0.09	33,460,579	1.18	68,427,926	2.42
LINE	758,982,393	26.81	551,346,462	19.47	680,109,554	24.02	1,018,625,838	35.98
LTR	130,528,749	4.61	6,744,768	0.24	84,503,020	2.98	141,521,674	5
SINE	279,268,337	9.86	0	0	63,907,283	2.26	324,361,957	11.46
Other	36,651,120	1.29	123	0	63,897,730	2.26	64,143,395	2.27
Unknown	496,273	0.02	0	0	227,519,165	8.04	228,015,202	8.05
Total	1,264,055,747	44.65	560,660,398	19.8	1,128,847,407	39.87	1,396,485,261	49.32

### Gene prediction and annotation

**Fig. 3:**
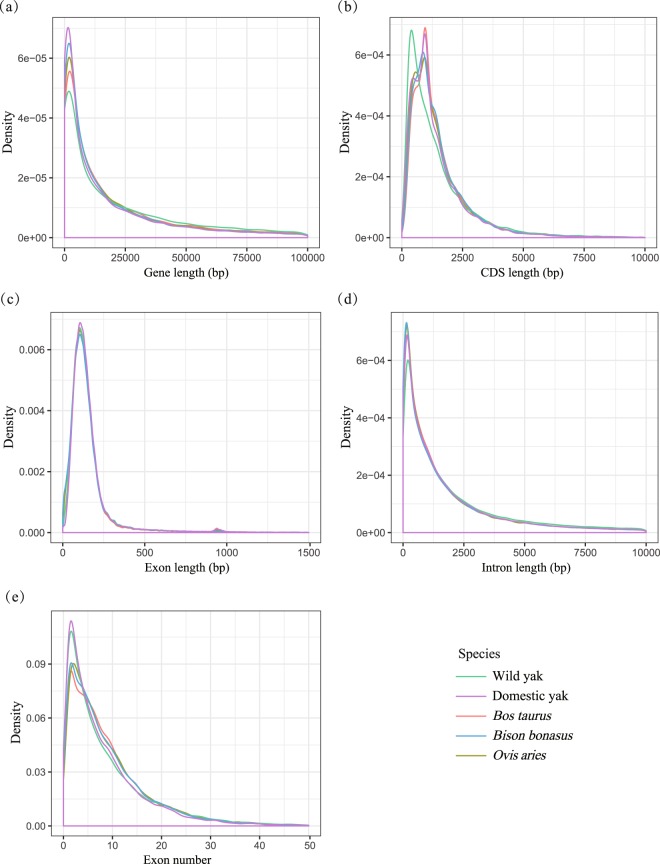
Comparison of structural characteristics of the wild yak genes with those of other mammals. (**a**) mRNA length, (**b**) CDS length, (**c**) Exon length, (**d**) Intron length, (**e**) Exon number per gene of wild yak, domestic yak, *Bos taurus* (UMD3.1), *Ovis aries* (Oar v3.1) and *Bison bonasus* (version 1.0). The *x*-axis represents length or number and the *y*-axis represents the density of genes.

We employed a combination of homology-based and *de novo* prediction methods to identify protein-coding genes. For homology-based prediction, protein sequences of seven species (*Bos taurus*, *Equus caballus*, *Homo sapiens*, *Ovis aries*, *Sus scrofa*, *Bison bonasus*, *Bos grunniens*) downloaded from Ensembl^18^ and GigaDB^19,20^ were aligned to the wild yak genome using TBLASTN^21^. Then GeneWise v2.4.1^22^ software was applied to search for accurately spliced alignments based on the filtered homologous genome sequences. For *de novo* prediction, we used Augustus^23^, Geneid^24^, GeneMark, GlimmerHMM^25^ and SNAP^26^ to predict genes with parameters trained on wild yak and human repeat-masked genomes. EVidenceModeler software (EVM, v1.1.1)^27^ was employed to generate a consensus gene set by integrating the genes predicted by the homology and *de novo* approaches. Low-quality genes of short length (proteins with fewer than 30 amino acids) and/or exhibiting premature termination were removed to produce the final gene set, which is composed of 22,910 genes (Fig. 3; Table 5).

**Table 5:** Summary statistics of predicted protein-coding genes in the wild yak genome.

Gene set	Total Genes Predicted	Average Gene Length (bp)	Average CDS Length (bp)	Average Exons per Gene	Average Exon Length (bp)	Average Intron Length (bp)
Augustus	21,211	50,645.17	1,531.32	9.04	169.39	6,096.22
Geneid	43,221	49,433.27	1,165.53	8.92	130.65	6,093.69
Genemark	159,214	1,442.59	356.34	2.52	141.29	713.63
GlimmerHMM	4,944	8,587.79	849.35	5.14	165.21	1,868.66
SNAP	120,892	11,877.32	799.25	5.36	149.13	2,541.06
*Bos taurus* (UMD3.1)	22,964	21,477.93	1,260.50	7.06	178.47	3,377.48
*Equus caballus* (EquCab2)	20,940	20,200.57	1,236.63	6.95	177.96	3,239.26
*Capra hircus* (ARS1)	23,252	24,941.52	1,303.75	7.25	179.78	3,834.31
*Homo sapiens* (GRCH38)	20,889	21,860.19	1,294.84	7.10	182.48	3,406.80
*Ovis aries* (Oar v3.1)	23,966	20,450.21	1,205.62	6.72	179.28	3,401.54
*Bison bonasus* (version 1.0)	23,422	22,271.56	1,279.32	7.20	177.62	3,422.69
*Bos grunniens* (version 1.1)	24,478	19,939.52	1,192.05	6.66	179.01	3,355.53
Final set	22,910	47,211.29	1,547.06	9.28	166.73	5,515.67

Putative biological functions of these predicted high-quality genes were assigned by searching against five publicly available databases: TrEMBL, Swiss-Prot^28^, InterPro^29^, Gene Ontology (GO) and Kyoto Encyclopedia of Genes and Genomes (KEGG)^30^. Approximately 90.18% of these genes were functionally annotated with at least one of these databases, with 90.05, 88.15, 83.51, 64.20 and 53.01% scoring positive hits in TrEMBL, SwissProt, InterPro, GO and KEGG, respectively (Table 6).

**Table 6:** Number of predicted genes in wild yak functionally annotated using indicated databases.

Database	Number	Percent(%)
Kegg	12,145	53.01
InterProScan	19,132	83.51
GO	14,709	64.2
Swissprot	20,195	88.15
Trembl	20,631	90.05
Annotated	20,660	90.18
Total Gene	22,910	—

## Data Records

The whole genome sequencing data were submitted to the NCBI Sequence Read Archive (SRA) database with accession number SRP194583 and Bioproject accession PRJNA531398^31^. The assembled draft genome of wild yak has been deposited at GenBank under the accession number of VBQZ00000000^32^. The annotation results of repeated sequences, gene structure and functional prediction were deposited in the Figshare database^33^.

## Technical Validation

### Quality assessment of the genome assembly

The assembly presented here is the first wild yak genome version. The contig N50 and scaffold N50 sizes were 63.2 kb and 16.3 Mb respectively, with the longest scaffold 75,900,441 bp. There are 258 scaffolds more than 1 Mb long, with a total length of 2,486,540,864 bp, representing 87.83% of the wild yak genome. By aligning the reads of short insert libraries to the wild yak assembly, we found more than 93.9% of the genome had >20-fold coverage, indicating high accuracy at the nucleotide level. BUSCO analysis carried out to assess the completeness of our assembly resulted in a BUSCO score of 96.8% (complete = 93.8%, single = 92.4%, duplicated = 1.4%, fragmented = 3.0%, missed = 3.2%, genes = 4,104). These results are comparable with those for the published European bison (wisent)^34^ and domestic yak^4^ genomes, suggesting our assembly has high quality and is quite complete.

### Gene prediction and annotation validation

Gene models in the wild yak assembly were predicted using a combination of homology-based and *ab initio* gene approaches. Then EVM software was employed to integrate the gene prediction results to produce a consensus gene set. To enhance the quality of the gene prediction, we removed low-quality genes of short length (proteins with fewer than 30 amino acids) and/or exhibiting premature termination. The final gene set consisted of 22,910 genes, and the distributions of gene length, CDS length, exon length, intron length and exon number were similar to those of other mammals (Fig. 3). BUSCO analysis was also performed to assess the completeness of these predicted genes, resulting in a BUSCO value of 97.7% (complete = 94.8%, single = 93.1%, duplicated = 1.7%, fragmented = 2.9%, missed = 2.3%, genes = 4,104) (Table 6). In addition, functional annotation of these predicted genes indicated that 90.18% of them could be assigned to at least one functional term (Table 5). These results clearly indicate that the annotated gene set of the wild yak genome is quite complete.

## Data Availability

The software versions, settings and parameters used are described below. (1) GCE, version 1.0.0, parameters used: kmer_freq_hash -k 21 -l reads.list -t 24 -i 5000000 -o 0 -p wild_yak & > kmer_freq.log; gce -f wild_yak.freq.stat -c 46 -g 155866493014 -m 1 -D 8 -b 1 > wild_yak.Table 2> wild_yak.log. (2) Lighter, version 1.1.1, parameters used: -k 17 3000000000 -trim -t 20. (3) Platanus, version 1.2.4, parameters used: platanus assemble -o wild_yak -f <insert size 280 bp pair-end reads> <insert size 500 bp pair-end reads> <insert size 800 bp pair-end reads> -t 30 -m 500 -tmp temp; platanus scaffold -o wild_yak -c wild_yak_contig.fa -b wild_yak_contigBubble.fa -IP1 < insert size 280 bp pair-end reads> -a1 280 -IP2 <insert size 500 bp pair-end reads> -a2 500 -IP3 < insert size 800 bp pair-end reads > -a3 800 -OP4 <insert size 2 k pair-end reads> -a4 2000 -OP5 <insert size 5 k pair-end reads> -a5 5000 -OP6 <insert size 10 k pair-end reads> -a6 10000; platanus gap_close -o wild_yak -c wild_yak_scaffold.fa -IP1 <insert size 280 bp pair-end reads> -IP2 <insert size 500 bp pair-end reads> -IP3 <insert size 800 bp pair-end reads> -OP4 < insert size 2 k pair-end reads > -OP5 <insert size 5 k pair-end reads> -OP6 <insert size 10 k pair-end reads>. (4) Gap Closer, version 1.12, parameters used: -l 150 -t 30, in configFile: max_rd_len = 100; Paired-end libs: reverse_seq = 0, asm_flags = 3; Mate-pair libs: reverse_seq = 11, asm_flags = 2. (5) BUSCO, version 3: mammal default parameters, mammalia_odb9. (6) BWA, version 0.7.15-r1140: default parameters. (7) SAMtools, version 1.3; default parameters. (8) RepeatMasker, version 4.0.7 (with RepBase library release-20170127). (9) RepeatModeler, RepeatModeler-open-1.0.11. (10) TRF, version 4.09, parameters used: trf wild_yak.gapclose.fa 2 7 7 80 10 50 500 -d -h. (11) TBLASTN, version 2.5.0, parameter used: –e 1E-5. (12) GeneWise, version 2.4.1, parameters used: -tfor/-trev (-rfor for genes on forward strand and -trev for reverse strand) -gff. (13) Augustus, version 3.2.3, parameter used: -species = human. (14) Geneid, version 1.0, parameters used: -3 -P. (15) Genemark, version 3.9, parameter used: -f gff3. (16) Snap, version 2006-07-28, parameter used: –gff. (17) GlimmerHMM, version 3.0.4, default parameters. (18) EVM, version 1.1.1, default parameters. (19) InterProScan, version 5.25-64.0, parameters: -f tsv -iprlookup -goterms -pa -t p.
